# Advances, Limitations and Future Challenges in the Management of Immunotherapy for Hematological Diseases and Solid Tumors

**DOI:** 10.3390/ijms24108812

**Published:** 2023-05-16

**Authors:** Pierre Tennstedt, Su Jung Oh-Hohenhorst

**Affiliations:** Martini-Klinik Prostate Cancer Center, University Medical Center Hamburg-Eppendorf, Martinistr. 52, 20246 Hamburg, Germany; s.oh-hohenhorst@uke.de

Our immune system is able to attack cancer cells by recognizing cellular mistakes and destroying them. To date, we still do not have sufficient knowledge on how well the immune system can fight degenerate cells that are developing. The idea of utilizing the patients’ own immune system to fight tumors has led to the remarkable progress in the field of cancer therapy in the last decade [1]. However, errors during the cell division process and multiple genetic mutations promoting oncogeneses allow the damaged cells to escape the immune system, which usually patrols and eliminates such cellular mistakes before they become uncontrollable. This Special Issue reviews diverse immunotherapeutic strategies, approved treatments and possible methods to predict therapeutic response. The experts in the field do not only summarize the recent breakthroughs and successful evidence in many types of cancers, but also the failures and the unexpected limitations in clinical practice. The contents should help us better understand the mechanisms of different immunotherapies and intend to discuss the challenges and opportunities in improving current strategies.

Capietto and colleagues have focused on the mutated peptide (“neoantigens”) induced by oncogenic events and presented on the histocompatibility complex (MHC) molecules to evoke potent anti-tumor immune responses [2]. The tumor-specific neoantigens can be generated from different sources: single-nucleotide variations or small insertions/deletion, gene fusions, alternative splicing variants and post-translational modifications. Since such cancer-specific neoantigens are known to induce potent immune response, numerous efforts to modulate the neoantigen expression, and different approaches to enhance the anti-tumor-specific T cell response are made [3,4]. Capietto and colleagues describe in their review the recent updates on the identification of neoantigens and prediction methods for therapeutic responses, as well as studies showing promising results for the DNA/RNA aberrations as novel sources of neoantigens [2]. One of the mentioned challenges was, for example, the identification and modulation of neoantigens arisen from non-coding DNA regions, e.g., long and short non-coding RNA transcripts (lncRNA) or pseudogenes. LncRNA influence the processes of regulation of transcription, splicing and translation, and pseudogenes can regain the lost protein-coding function in tumor cells. Moreover, the T-cell response varies with the type of neoantigens or targeting approaches, and may not be sufficient enough to fight the tumor cells effectively. The improvement of antigen quality and overcoming some manufacturing challenges requires more studies in this field.

From a therapeutic perspective, certain dysregulated kinase signaling cascades found in tumors, which could mediate different immune responses by targeting, are discussed by Kim and colleagues [5]. Among others, the Rho-kinase (ROCK) pathway is known to promote tumor cell progression, migration, metastasis, and extracellular matrix remodeling. ROCK is a serine/threonine kinase [6] and has two isoforms, ROCK1 and ROCK2, which are differentially expressed in specific tissues [7]. In tumors, an increasingly stronger expression of Rho-K with tumor progression has been observed [5]. There are already two clinically approved selective ROCK-inhibitors, belumosudil and netarsudil. In addition, the Phase 1 clinical trial of AT13148, the first dual potent ROCK-AKT kinase (AKT) inhibitor for the treatment of advanced solid tumor has currently been completed. Concerning its role in immunotherapy, ROCK is considered to be an effective modulator of immune cells due to the observed activation of several immune cells (dentritic cells, T cells, NK-cells ect.) and phagocytosis during the inhibition of ROCK-pathways. Based on the observation that the blockade of ROCK in cancer cells was able to evoke sequential immune cell responses, the therapeutic effects of ROCK-inhibitors could be exponentiated with other combined chemotherapeutic agents. However, most studies on ROCK in the field of cancer therapy have been focused on the direct effectiveness of ROCK on cancer cells, rather than its surrounding components, so further functional studies about the immunomodulating role of ROCK should be performed.

Although numerous potential targets and novel pharmacologic compounds for immunotherapy have been evaluated in recent years, progress in the establishment of predictive biomarkers for the therapy response and patient selection was limited. In this respect, emerging strategies to select patients who can benefit from immunotherapy and the screening methods are also discussed in this Special Issue.

Amato and colleagues suggest microsatellite instability (MSI) as a predictive biomarker for therapy response to immunotherapy [8]. MSI has been observed in many tumors, particularly in colorectal cancer. MSI can arise due to mutations, hypermethylation, and epigenetic alteration by miRNA in the genes of the mismatch repair system. Tumors with MSI have a high level of cytotoxic T lymphocytes and increased expression of the checkpoint proteins CTLA4, PD-1, PD-L1, LAG-3, and IDO [9,10], which felicitate a better response to immunotherapy. Hence, the detection of MSI is an accepted biomarker for the selection of patients who are to be treated with immune checkpoint inhibitors [11]. The review of Amato and colleagues provides an overview of MSI in various cancers and highlights its potential predictive/prognostic role, as well as the related clinical trials. Moreover, different assay used to detect MSI in clinical practice (immunohistochemistry, polymerase chain reaction and next-generation sequencing) are compared. The current time and cost-consuming detection methods should still be optimized and meet legal standards and medical guidelines for uniform recommendations. However, the identification and screening of additional specific tumor antigens in each individual patient may facilitate more precise stratification for patients, as well as monitoring during immunotherapy.

Immunotherapy might change the paradigm of therapeutic management of hematologic malignancies of the past two decades, particularly the acute lymphoblastic leukemia (ALL), by multiple novel immunotherapies. Despite the mentionable success in the B-cell and T-cell ALL or anaplastic large cell lymphoma in the primary or secondary (in the case of recurrence) therapy setting, the response to immunotherapeutic treatment cannot be guaranteed at the time point of therapy initiation, and it is associated with a relatively high rate of relapse. The authors Newman and Teaschey describe the challenges of translating immunotherapies for pediatric patients with T-cell malignancies, which were not expected initially [12]. These include therapy resistances, complexities of the self-killing of the chimeric antigen receptor (CAR) T-cells, risk of product contamination with malignant cells, the potential toxicity of T-cell aplasia, graft versus host disease and immunotherapy side effects, such as cytokine release syndrome. Despite these hurdles, several promising strategies to overcome these challenges for T-cell malignancies are under evaluation, among others, cluster of differentiation (CD) antigens, which are found on the cell surfaces of, e.g., leukocytes, and recognized by the immune system. Due to increased CD-20-positive B lymphocytes in the blood of patients, treatment with a human/murine chimeric anti-CD20 monoclonal antibody could be established as standard therapy [13]. The next stage of development includes antibodies conjugated to a chemotherapeutic agent and bispecific T-cell engagers, which can be applied to patients with hematological diseases characterized by increased CD-33, CD-22 or CD-19 positive cells. In addition, a promising development can also be observed for the CAR cell therapies of T cells, natural killer cells and macrophages. The efforts to overcome the current limitations should lead to the improvement of immunotherapy in T-cell malignancies, which may someday be incorporated in up-front protocols in order to prevent relapses.

The “cold tumor” phenomenon is one of the problems that must be solved in solid tumors, e.g., in prostate cancer (PCa). This is associated with a restricted immunotherapeutic response due to an immunosuppressive tumor microenvironment (TME). In the extensive review by von Amsberg et al., the difficulties and innovative solutions of immunotherapy are described, together with an extensive overview of the current status of clinical studies in PCa [14]. To date, the established immunotherapies for melanoma, renal cell carcinoma or lung cancer have been shown to be insufficient in treating PCa. Moreover, the planning and implementation of immunotherapy for PCa seems to be more complex than expected. Among others, the significantly lower PD-1/PD-L1 expression levels, defected DNA damage repair genes, low frequency of microsatellite instability and PTEN inactivation/deletion, which are closely associated with an immunosuppressive TME, could reduce the success of possible immunotherapies. The current immunotherapeutic treatment strategies for prostate cancer are based on the activation of the immune system and the targeted combating of tumor cells with increased expression of the tumor-associated antigens, such as PSA, PSMA, PAP or the prostate stem cell antigen (PSCA). In this context, several types of vaccines are currently being evaluated. Immunotherapies with checkpoint inhibitors against CTLA-4, PD-1/PD-L1 or PARP can also treat the tumor successfully, but only in well-selected and molecular genetically characterized patients. Since targeting androgen receptor (AR) signaling is able to affect the immune system directly, von Amsberg and colleagues mention that immunotherapeutic agents, such as the checkpoint inhibitor PD-1/PD-L1, may induce stronger anti-cancer effects in combination with other therapeutic strategies, such as AR-axis-targeting therapies, chemotherapies, including PARP- and tyrosine kinase inhibitors and radiation. The results of the studies on various combinations of checkpoint inhibitors with other approved PCa therapy options are eagerly awaited. The application of bispecific T Cell Engagers (BiTEs), the synthetic proteins designed to activate T cells and the transfusion of the chimeric antigen receptor T cells (CAR-T Cells), which are genetically modified T cells transfected with a chimeric antigen receptor directed against a tumor antigen, belong to the new promising treatment strategies. Ongoing therapies target overexpressed tumor proteins, such as PSMA, ADAM17, PSCA, delta-like ligand 3 (DLL3), or kallikrein 2 (KLK2). Despite the unexpectedly severe toxicities and moderate therapeutic success, which should definitely be improved in the near future [15], the growing knowledge on the specific immunosuppressive milieu of PCa and possible counter-regulatory interventions give great hope that PCa patients would also benefit from immunotherapy in the future.

Overall, immunotherapy represents an effective therapeutic option for many solid tumors and hematological diseases and could even offer the possibility of combating advanced tumors and metastases in the near future. Despite such notable advances in this field, there are still severe limitations and challenges that should definitely be optimized. Future work must be directed towards improve accessibility to and the therapeutic effects of novel agents, as well as establishing tools for response assessment and better patient stratification.
